# The Emergence of Rift Valley Fever in Gedaref State Urges the Need for a Cross-Border One Health Strategy and Enforcement of the International Health Regulations

**DOI:** 10.3390/pathogens10070885

**Published:** 2021-07-13

**Authors:** Ayman Ahmed, Iman Mahmoud, Mawahib Eldigail, Rehab M. Elhassan, Scott C. Weaver

**Affiliations:** 1 Institute of Endemic Diseases, University of Khartoum, Khartoum 11111, Sudan; 2 World Reference Center for Emerging Viruses and Arboviruses, University of Texas Medical Branch, Galveston, TX 77550, USA; sweaver@utmb.edu; 3 Institute for Human Infections and Immunity, Department of Microbiology and Immunology, University of Texas Medical Branch, Galveston, TX 77550, USA; 4 National Public Health Laboratory, Sudan Federal Ministry of Health, Khartoum 11111, Sudan; imansust@hotmail.com (I.M.); menana_555@hotmail.com (M.E.); rehab-2008@live.com (R.M.E.)

**Keywords:** Rift Valley fever virus (RVFV), emergence, epidemic, outbreak, arboviruses, zoonotic diseases, one health, Gedaref state, Sudan

## Abstract

This study investigated the causative agent of a haemorrhagic fever epidemic in Gedaref state, south-east Sudan. Six cases of febrile illness with haemorrhagic manifestations presented at outpatient health-clinics. Blood samples were collected from the patients and shipped to Khartoum where they were tested for dengue virus (DENV), chikungunya virus (CHIKV), and Rift Valley fever virus (RVFV) using real-time qPCR. Fifty percent (3/6) of them tested positive for RVFV and neither DENV or CHIKV was detected. All patients were males between 20 and 48 years old who had no history of recent travel. This finding describes the first emergence of RVFV in Gedaref state. Considering that the state hosts a major market of livestock, and it has one of the largest-seasonal open pastures in the country that is usually flooded with herds from the neighbouring states and countries during the rainy season, this emergence could represent a major threat to public health in the region and countries importing animals and/or animal products from east Africa. Therefore, we urge the policymakers of the health and animal resources sectors to implement a one health strategy with a well-established early warning surveillance and response system to prevent the establishment of the disease in the area.

## 1. Introduction

Rift Valley fever (RVF) is a zoonotic viral haemorrhagic disease that affects the health and well-being of both humans and animals. The disease is caused by the arthropod-borne virus (arbovirus) RVF phlebovirus (RVFV), which is a member of the *Phenuiviridae* viral family [1]. RVFV was first isolated and characterized following a large-scale epizootic in Kenya in 1931 [2]. Several mosquito species are competent vectors of RVFV, differently contributing in the virus transmission in both the enzootic cycle among sylvatic animals and the epizootic/epidemic transmission cycle among domestic animals and people [2]. Additionally, RVFV infection can be acquired through close contact with infected animals, and through contact with the raw milk or meat from infected animals [3]. Furthermore, vertical transmission from mother to offspring has been documented in humans and livestock as well as in several genera of mosquitoes [3].

Several parasitic diseases are endemic to Sudan such as malaria, leishmaniasis, and onchocerciasis [4,5,6]. More importantly, many major human and zoonotic arboviral diseases are also endemic to the country, with increasingly serious outbreaks, including chikungunya [7], dengue [8], West Nile (WN), Crimean–Congo haemorrhagic fever (CCHF) [9], yellow fever and RVF [1,3]. Recent studies have highlighted the geographical expansion, increased rate and intensity of these outbreaks, and the overall change in the burden and epidemiologic features of these arboviral diseases in the country. Furthermore, some of these changes were attributed to risk factors such as conflicts and high population dynamics between endemic disease areas and areas without endemic disease [1,8,9,10], climate change [1,7], globalization and unplanned urbanization [8,10], and the extremely limited diagnostic capacity and lack of practicable health policy for the prevention and control of arboviruses in the country [3,11,12].

RVFV is enzootic in Sudan with frequent epizootic/epidemic emergence events [1,3]. The circulation of the RVFV in Sudan was first reported in 1936 [3]. In addition to causing abortion among animals, infection with RVFV was found to be strongly correlated with miscarriage among women [13]. Furthermore, in addition to the public health risk, RVF has devastating impacts on the economy and food security, particularly among the poor communities and countries that rely heavily on domestic animal and animal product exportation such as Sudan [14].

In this short communication, we document the emergence of RVFV in Gedaref state, south-east Sudan, that shares borders with both Eritrea and Ethiopia, and report the first cases of RVF in the area.

## 2. Results

The molecular investigation revealed that three of the six patients (50%) who presented with viral haemorrhagic fever to the outpatient clinics were infected with RVFV. The clinical presentations of the patients included 100% with fever and bleeding, and 67% with headache. The demographics of the patients showed that all were male aged between 20 and 48 years of age (Table 1). None of the patients had a recent history of travel outside of Gedaref state.

## 3. Discussion

This is the first ever documentation of RVFV infection in Gedaref state, as none of the previous molecular or serological studies have detected the virus or reported previous exposure in the area [3]. Six cases of haemorrhagic fever were presented to outpatient healthcare clinics in Gedaref state in late October 2019. Our molecular analysis confirmed RVFV infection in three cases, or 50% of the tested blood samples. However, it is very likely that our study only identified the severe cases with haemorrhagic disease and missed the mild and/or asymptomatic infections, particularly because many cases are mild or asymptomatic and previous studies suggest that the actual prevalence of RVF is higher than that reported during epidemics [8,15,16]. There are many risk factors that might influence the emergence of arboviral diseases in Sudan, including climate change [1,7], conflicts [8,9], increased local and international human movements [10], as well as trade, unplanned urbanization, and socioeconomic status of the individuals and communities in general [17,18,19].

In 2019, Sudan witnessed two major unusual events related to arboviral diseases. The first was the massive outbreak of CHIKV infection with more than 47,000 cases largely reported from the two states, Red Sea and Kassala, and the neighbouring Gedaref state [3,7]. The second event was the occurrence of a unique outbreak of RVF north of the country, River Nile state, which unusually occurred before the rainy and typical transmission season of vector-borne diseases in the country [1]. These serious outbreaks were coincident with a significant security event in Sudan at that time: the revolution which ended more than 30 years of dictatorship governance. This revolution might have accidentally contributed to the development of these events, because there was a partial or complete impairment of civil services including the health system, with its crucial components such as healthcare, diagnostics, case management (hospitalization) and vector surveillance and control [1]. Moreover, the violence associated with the revolution caused massive human population movement that might have caused people to move from endemic to non-endemic areas and vice versa, leading to the introduction of native pathogens and reducing local herd immunity, respectively [1].

Poor resources and limited health systems are major challenges for the early detection, prevention and control of arboviral disease epidemics in the country [3]. The fact that 50% (3/6) of the patients who presented with haemorrhagic febrile illness tested negative for the major suspected arboviruses, CHIKV, DENV, and RVFV, suggests that other aetiologies of haemorrhagic fever might be circulating in the area, particularly because Sudan is geographically and environmentally prone to the transmission and epidemics of several haemorrhagic fevers [20,21]. However, due to the limited diagnostic capacity in the country and the suspected cases of haemorrhagic fever, arboviral diseases infections are only laboratory-tested in the National Public Health Laboratory using limited options of commercially available kits annually provided by the World Health Organization (WHO). This limitation on differential diagnoses and choice of laboratory tests based on the available kits is exacerbated by the high proportion of haemorrhagic fever cases that are left undifferentiated, even for known aetiologic agents during outbreak investigations [1,8,9,10]. These limitations in the diagnostic capacity in Sudan are further exacerbated by the limited local knowledge about these diseases, including the lack of updates to case definitions that could improve differential diagnoses. Establishing in-country viral genome sequencing could provide a robust and sensitive diagnosis for the many cases of unidentified febrile illness and generate valuable evidence and information about the dynamics, locally circulating strains, and identities of emerging viruses [3,9].

Several factors make the expansion and emergence of RVFV that we report in new areas of high importance and very alarming, not only for Sudan, but particularly for the neighbouring countries with open borders with Gedaref: namely Eritrea and Ethiopia. Additionally, RVFV is not only threatening global public health but also national security, agricultural and food safety, and the socioeconomic stability of the communities at risk. Therefore, RVF has been classified by the U.S. Center for Disease Control and Prevention (CDC) as a potential agent of bioterrorism. Moreover, the Global Alliance for Vaccines and Immunizations (GAVI) suggests that the next pandemic might be caused by RVF [22]. Due to its threat to public health and the high risk of epidemics, the WHO identifies RVFV as a priority pathogen for its research and development blueprint [23]. Therefore, improving RVF diagnostic capacity in endemic countries and countries at risk of epidemic emergence is crucial to prevent further emergence and help in responding to outbreaks [22,24].

Considering the spatial and temporal dimensions of the RVFV emergence we describe, additional risks need to be addressed and mitigated. Around 80,000 Ethiopian seasonal migrants and temporal workers cross the borders to work on Sudanese farms in Gedaref state annually during the rainy season [25]. Additionally, this year, according to the United Nations High Commission for Refugees (UNHCR), around 63,000 Ethiopian refugees along with their animals arrived in Gedaref state to escape the Tigray war that has flared up recently in the Ethiopia Tigray region, on the borders with Eritrea and Sudan, seeking for safety, shelter, and food in Sudan. These refugees are currently housed in humanitarian camps in Gedaref state and many more thousands are expected to arrive by the end of 2021 (http://unhcrinfo.org/sudan/, accessed on 20 June 2021). An epidemiological study to investigate the seroprevalence of RVFV among the human, livestock, and wild animal populations in the area would generate essential information about the disease transmission in the area and estimate the extent of its public health and economic threat [26]. Moreover, with the movement of human and animal populations across the international borders along with international humanitarian responders traveling to and from to several other countries, strict enforcement of the International Health Regulations (IHRs) is critical for limiting further importation and/or exportation of the RVFV through travellers [27].

An additional risk is that Gedaref state hosts a major market for livestock that is mainly active before the holy Haj season, around July/August, when Muslims globally buy sheep to slaughter them for religious rituals. This represents similar circumstances to those in in 2000 which led to the first introduction of RVFV into the Arabian Peninsula from East Africa [16]. Therefore, better intergovernmental coordination, particularly between ministries of health and agriculture, including the institutionalization and practical implementation of a one health strategy, are essential for the prevention, early detection, and control of the RVFV and other zoonotic pathogens in the area prior to the development of epidemics [11,28,29]. Although it might seem costly to coordinate the proper implementation of a One Health strategy among the IHRs of the three neighbouring countries who share open borders, potential scenarios suggest the devastating impacts of a large-scale epidemic among these agriculturally dependent poor and fragile communities in the region. Additionally, the high risk of exporting RVFV to other regions and/or countries is real and requires the engagement of all global stakeholders, including private and public organizations, at the preparedness and response levels [1,3,11]. Several components of a one health strategy might be readily available for application, including limiting the movement of livestock between the RVF-endemic and non-RVF-endemic areas, making the currently available vaccine against RVF (for use on animals) accessible to herd owners, raising the awareness of herd owners about the interconnected health of humans and animals, and how to live safely together. Additionally, implementing national surveys to determine the diversity, transmission, and risks associated with the emergence of zoonotic diseases, and periodically investigating the environmental suitability for emergence, are needed [30]. In the long term, establishing a molecular surveillance system for the detection of pathogens in vector populations prior to their emergence among humans and animal populations, and developing a national institute in charge of implementing the one health strategy, might be useful for the early detection and responses to emerging zoonotic diseases. Moreover, governments, donors, and funders should support the Coalition for Epidemic Preparedness Innovations (CEPI) in their pursual of developing a vaccine against RVFV that is safe for human use [31]. Previous experiences of Sudan with RVF outbreaks show that they were rarely confined to a single region of the country [1], but instead rapidly spread over many regions of the country with infections reported among both humans and animals, and rapidly expand beyond the limited responses of the Ministry of Health [1,11,28,29]. This underscores the need for establishing a countrywide early warning and response system (EWARS).

Due to the ongoing COVID-19 pandemic, the health system in Sudan has further deteriorated and most of the prevention and control measures for vector-borne diseases have been interrupted. This could lead to several serious outbreaks of other diseases in the area, particularly since Ethiopia is one of the African countries most strongly affected by COVID-19, and Gedaref is one of the most affected Sudanese States [32]. Therefore, carefully strategized preparedness measures like increasing diagnostic capacity, and raising the awareness of the public health sector, healthcare providers, and communities at risk about the routes of transmission, personal protection measures, and clinical symptoms of arboviral diseases is needed. Additionally, policymakers should be engaged for resource mobilization and support to ensure interministerial coordination. Additionally, the public health system and healthcare providers for both humans and animals need to be trained on essential topics including proper documentation, timely reporting, case definitions and differential diagnoses, as well as outbreak investigations [1,11]. In particular, the lack of proper documentation and reporting of health information, mainly during health emergencies and epidemics, is greatly compromising decision and policy making as well as misguiding healthcare providers [11]. This results in delays in the recognition and response to arboviral disease epidemics [1,3,7,8,9,10].

## 4. Materials and Methods

### 4.1. Study Design

This was a retrospective–descriptive study analysing secondary data that were collected during the investigation of a haemorrhagic fever epidemic. Blood samples were collected from patients presenting with haemorrhagic fever of unknown nature to outpatient clinics. Samples were shipped to the Sudan National Public Health Laboratory in Khartoum, where they were tested for the major human haemorrhagic arboviruses CHIKV, DENV, and RVFV using real-time qPCR with the commercially available kits, RealStar^®®^ Chikungunya RT-PCR Kit 2.0, RealStar^®®^ Dengue Virus RT-PCR Kit 1.0, and RealStar^®®^ Rift Valley Fever Virus RT-PCR Kit 1.0 (Altona Diagnostics GmbH, Hamburg, Germany) according to the manufacturer’s instructions. Ideally, at least the negative samples would have been tested for other causative agents of haemorrhagic fever and/or testing the immunological response by IgM-ELISA. However, due to the limited resources in the country, the decision for a diagnostic test to be performed is largely influenced by the availability of kits, techniques, and additional resources [3].

### 4.2. Study Area

**Figure 1:**
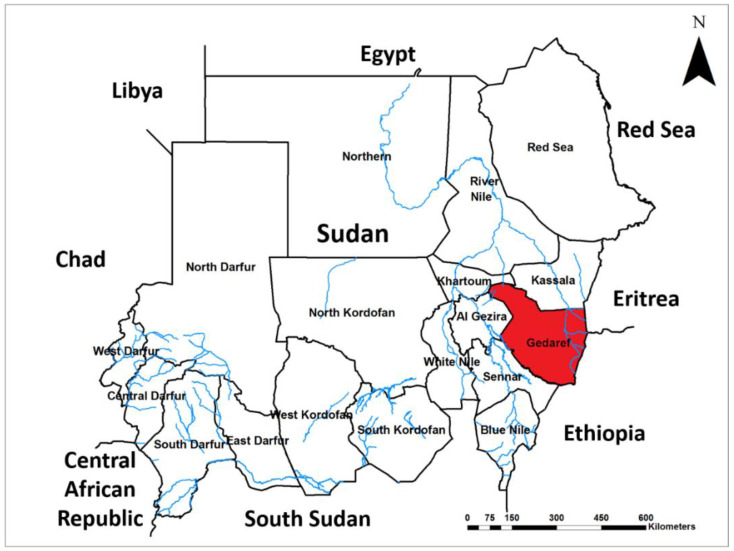
Map of Sudan shows the study site: Gedaref state is highlighted in red.

Gedaref is one of the 18 states of Sudan and is located in the south-eastern part of the country at roughly 14°0′ N and 35°0′ E coordinates. The state is mainly characterized by widely distributed, rich savannahs and valleys, with heavy seasonal rains between August through October, averaging at 800 mm in annual precipitation [33]. The total human population of 1,827,181 individuals lives in an area of 75,263 km². Most are nomads, internally displaced persons (IDPs), and refugees coming from the neighbouring countries of Eritrea, Ethiopia, and from West African countries, attracted by the large open pasture spaces. However, farming, animal breeding, and trading are the main sources of income. More importantly, the state seasonally hosts around 7 million in livestock during and after the rainy season, as long as the seasonal pastures are available (Figure 1).

### 4.3. The Outbreak Investigation

By the end of the rainy season of 2019, in late October, six patients presented to outpatient healthcare clinics in the Gedaref state. However, due to the lack of molecular diagnostic tools in most of the rural and remote areas of Sudan [10], blood samples from those patients were collected and sent to the National Public Health Laboratory in Khartoum. Samples were tested for the causative viruses of the three most prevalent arboviruses in the country: dengue, chikungunya, and RVF viruses, using rt-qPCR and the commercially available kits (Altona Diagnostics GmbH, Hamburg, Germany).

## 5. Conclusions

Institutionalization of the one health strategy and early warning and response surveillance systems for emerging and endemic arboviruses are urgently needed in Sudan for the prevention and control of arboviral diseases and other haemorrhagic fevers. Additionally, the government, stakeholders related to human and animal health and food security, biomedical and health research institutes, and global partners are urged to support investments in improving the health and research status of the country to prevent further epidemics. Particular attention is needed for improving the diagnostic capacity throughout the country, prioritising areas where frequent outbreaks of arboviruses are occurring. We recommend strengthening the implementation of International Health Regulations to prevent the exportation and importation of pathogens and vectors, and the establishment of molecular surveillance for the detection of pathogens among vector populations prior to the infection of humans and livestock.

## Figures and Tables

**Table 1:** The general characteristics of the viral haemorrhagic fever cases.

Character		Frequency	Percentage/Average
Sex	Male	6	100%
	Female	0	0
Age range	20–48 years	6	30 years
Symptoms	Fever	6	100%
	Bleeding	6	100%
	Headache	4	67%
Laboratory results for RVFV	Positive	3	50%
	Negative	3	50%

## Data Availability

All data used for this study are included in this published article.

## References

[1] Ahmed A., Ali Y., Elduma A., Eldigail M.H., Mhmoud R.A., Mohamed N.S., Ksiazek T.G., Dietrich I., Weaver S.C. (2020). Unique Out-break of Rift Valley Fever in Sudan, 2019. Emerg. Infect. Dis..

[2] Gaudreault N.N., Indran S.V., Balaraman V., Wilson W.C., Richt J.A. (2018). Molecular aspects of Rift Valley fever virus and the emergence of reassortants. Virus Genes.

[3] Ahmed A., Dietrich I., LaBeaud A.D., Lindsay S.W., Musa A., Weaver S.C. (2020). Risks and Challenges of Arboviral Diseases in Sudan: The Urgent Need for Actions. Viruses.

[4] Mohamed N.S., Ali Y., Muneer M.S., Siddig E.E., Sibley C.H., Ahmed A. (2021). Malaria epidemic in humanitarian crisis settings the case of South Kordofan state, Sudan. J. Infect. Dev. Ctries..

[5] Duong V., Lambrechts L., Paul R.E., Ly S., Lay R.S., Long K.C., Huy R., Tarantola A., Scott T.W., Sakuntabhai A. (2015). Asymptomatic humans transmit dengue virus to mosquitoes. Proc. Natl. Acad. Sci. USA.

[6] Ahmed A., Elbashir A., Mohamed A.A., Alim A.A., Mubarak A., Abdelrahman D., Mohammed E., Mohamed N.S., Elaagip A.H., Zarroug I.M.A. (2020). Socioeconomic impacts of elimination of onchocerciasis in Abu-Hamed focus, northern Sudan: Lessons after elimination. BMC Res. Notes.

[7] Ahmed A., Ali Y., Mohamed N.S. (2020). Arboviral diseases: The emergence of a major yet ignored public health threat in Africa. Lancet Planet. Health.

[8] Ahmed A., Ali Y., Elmagboul B., Mohamed O., Elduma A., Bashab H., Mahamoud A., Khogali H., Elaagip A., Higazi T. (2019). Dengue Fever in the Darfur Area, Western Sudan. Emerg. Infect. Dis..

[9] Ahmed A., Elduma A., Magboul B., Higazi T., Ali Y. (2019). The First Outbreak of Dengue Fever in Greater Darfur, Western Sudan. Trop. Med. Infect. Dis..

[10] Ahmed A., Eldigail M., Elduma A., Breima T., Dietrich I., Ali Y., Weaver S.C. (2021). First report of epidemic dengue fever and malaria co-infections among internally displaced persons in humanitarian camps of North Darfur, Sudan. Int. J. Infect. Dis..

[11] Ahmed A. (2020). Urgent call for a global enforcement of the public sharing of health emergencies data: Lesson learned from serious arboviral disease epidemics in Sudan. Int. Health.

[12] Elduma A.H., LaBeaud A.D., APlante J., Plante K.S., Ahmed A. (2020). High Seroprevalence of Dengue Virus Infection in Sudan: Systematic Review and Meta-Analysis. Trop. Med. Infect. Dis..

[13] Baudin M., Jumaa A.M., Jomma H.J.E., Karsany M.S., Bucht G., Näslund J., Ahlm C., Evander M., Mohamed N. (2016). Association of Rift Valley fever virus infection with miscarriage in Sudanese women: A cross-sectional study. Lancet Glob. Health.

[14] Hassan O.A., Ahlm C., Sang R., Evander M. (2011). The 2007 Rift Valley Fever Outbreak in Sudan. PLoS Negl. Trop. Dis..

[15] Ahmed A. (2021). Current Status of Mosquito-Borne Arboviruses in Sudan, and Challenges of Surveillance and Responses [Internet]. Online. https://www.malariaconsortium.org/pages/webinars/webinar-mosquito-borne-arboviruses-the-rising-global-threat.htm.

[16] Shoemaker T., Boulianne C., Vincent M.J., Pezzanite L., Al-Qahtani M.M., Al-Mazrou Y., Khan A.S., Rollin P.E., Swanepoel R., Ksiazek T.G. (2002). Genetic Analysis of Viruses Associated with Emergence of Rift Valley Fever in Saudi Arabia and Yemen, 2000–2001. Emerg. Infect. Dis..

[17] Chevalier V., Pépin M., Plée L., Lancelot R. (2010). Rift Valley fever—A threat for Europe?. Eurosurveillance.

[18] Elaagip A., Alsedig K., Altahir O., Ageep T., Ahmed A., Siam H.A., Samy A.M., Mohamed W., Khalid F., Gumaa S. (2020). Seroprevalence and associated risk factors of Dengue fever in Kassala state, eastern Sudan. PLoS Negl. Trop. Dis..

[19] Whiteman A., Loaiza J.R., Yee D.A., Poh K.C., Watkins A.S., Lucas K.J., Rapp T.J., Kline L., Ahmed A., Chen S. (2020). Do socioeconomic factors drive Aedes mosquito vectors and their arboviral diseases? A systematic review of dengue, chikungunya, yellow fever, and Zika Virus. One Health.

[20] Pigott D.M., Deshpande A., Letourneau I., Morozoff C., Reiner R.C., Kraemer M.U.G., Brent S.E., Bogoch I.I., Khan K., Biehl M.H. (2017). Local, national, and regional viral haemorrhagic fever pandemic potential in Africa: A multistage analysis. Lancet.

[21] Drosten C., Göttig S., Schilling S., Asper M., Panning M., Schmitz H., Günther S. (2002). Rapid Detection and Quantification of RNA of Ebola and Marburg Viruses, Lassa Virus, Crimean-Congo Hemorrhagic Fever Virus, Rift Valley Fever Virus, Dengue Virus, and Yellow Fever Virus by Real-Time Reverse Transcription-PCR. J. Clin. Microbiol..

[22] (2021). GAVI TGA for V and I. The Next Pandemic: Rift Valley Fever?. https://www.gavi.org/vaccineswork/next-pandemic/rift-valley-fever.

[23] The World Health Organization (2021). Prioritizing Diseases for Research and Development in Emergency Contexts. https://www.who.int/activities/prioritizing-diseases-for-research-and-development-in-emergency-contexts.

[24] Pedarrieu A., Mellouli F.E., Khallouki H., Zro K., Sebbar G., Sghaier S., Madani H., Bouayed N., Lo M.M., Diop M. (2021). External quality assessment of Rift Valley fever diagnosis in countries at risk of the disease: African, Indian Ocean and Middle-East regions. PLoS ONE.

[25] Clark M.H.A., Warimwe G.M., Nardo A.D., Lyons N.A., Gubbins S. (2018). Systematic literature review of Rift Valley fever virus se-roprevalence in livestock, wildlife and humans in Africa from 1968 to 2016. PLoS Negl. Trop. Dis..

[26] Cleton N., Koopmans M., Reimerink J., Godeke G.-J., Reusken C. (2012). Come fly with me: Review of clinically important arboviruses for global travelers. J. Clin. Virol..

[27] (2018). EUTF for Africa TEUETF for Stability and Addressing Root Causes of Irregular Migration and Displaced Persons in A. Sudan and Ethiopia Met for the First Time to Discuss Improvements to Seasonal Labour Migration. EU Emergency Trust Fund for Africa—European Commission. https://ec.europa.eu/trustfundforafrica/all-news-and-stories/sudan-and-ethiopia-met-first-time-discuss-improvements-seasonal-labour_en.

[28] Hassan O.A., Affognon H., Rocklöv J., Mburu P., Sang R., Ahlm C., Evander M. (2017). The One Health approach to identify knowledge, attitudes and practices that affect community involvement in the control of Rift Valley fever outbreaks. PLoS Negl. Trop. Dis..

[29] Hassan O.A., Ahlm C., Evander M. (2014). A need for One Health approach—Lessons learned from outbreaks of Rift Valley fever in Saudi Arabia and Sudan. Infect. Ecol. Epidemiol..

[30] Bashir R.S.E., Hassan O.A. (2019). A One Health perspective to identify environmental factors that affect Rift Valley fever transmission in Gezira state, Central Sudan. Trop. Med. Health.

[31] Petrova V., Kristiansen P., Norheim G., Yimer S.A. (2020). Rift valley fever: Diagnostic challenges and investment needs for vaccine development. BMJ Glob. Health.

[32] Ahmed A., Mohamed N.S., El-Sadig S.M., Fahal L.A., Abelrahim Z.B., Ahmed E.S., Siddig E.E. (2021). COVID-19 in Sudan. J. Infect. Dev. Ctries..

[33] Zarroug I.M.A., Elaagip A., Gumaa S.G., Ali A.K., Ahmed A., Siam H.A.M., Abdelgadir D.M., Surakat O.A., Olamiju O.J., Boakye D.A. (2019). Notes on distribution of Simulium damnosum s. l. along Atbara River in Galabat sub-focus, eastern Sudan. BMC Infect. Dis..

